# Schwannoma of the larynx

**DOI:** 10.1186/1758-3284-1-24

**Published:** 2009-07-08

**Authors:** Jörg Ebmeyer, Ulf Reineke, Hans-Björn Gehl, Ulrich Hamberger, Robert Mlynski, Matthias Essing, Tahwinder Upile, Holger Sudhoff

**Affiliations:** 1 Department of Otorhinoloaryngology, Head and Neck Surgery, Klinikum Bielefeld, Academic Teaching Hospital University of Münster, Bielefeld, Germany; 2 Department of Diagnostic and Interventional Radiology, Klinikum Bielefeld, Academic Teaching Hospital University of Münster, Bielefeld, Germany; 3 Department of Pathology, Klinikum Bielefeld, Academic Teaching Hospital University of Münster, Bielefeld, Germany; 4 Department of Otorhinoloaryngology, Head and Neck Surgery, University Hospital of Würzburg, Germany; 5 ENT practice, Bielefeld, Germany; 6 The University College London, Ear Institute, Gray's Inn Road, London, UK

## Abstract

**Objectives:**

Neurogenic tumors of the larynx are extremely rare. The goal of this report is to advert to this rare disease, to review and discuss diagnostics, differential diagnoses and treatment options. Study Design: Retrospective case report and review of the literature. Methods: Case report of a schwannoma of the supraglottic larynx and review of the English- and German-language literature regarding neurogenic tumors of the larynx. Results: Neurogenic laryngeal tumors typically involve the supraglottic larynx, rarely the glottis. They can course globus sensation, dysphagia, dysphonia and upper airway obstruction. Imaging does not yield a definite diagnosis. The only curative treatment option is complete surgical resection. Conclusions: A definite diagnosis can only be made histologically. Endoscopic (laser-) resection for smaller lesions and external approaches for larger lesions are recommended treatment options.

## Introduction

About 45% of all neurogenic tumors occur in the head and neck region and are mostly located in the parapharyngeal space [1,2]. Two types of neurogenic tumors must be distinguished: Schwannomas and neurofibromas. Schwannomas emanate from perineural Schwann cells, and are well encapsulated, growing adjacent to the parental nerve but extrinsic to the nerve fascicles [2]. Neurofibromas on the other hand derive from perineural fibrocytes, and are not encapsulated and are usually intertwined with the parental nerve fascicles [2,3]. Multiple neurofibromas are observed in Neurofibromatosis.

The location of Schwannoma or neurofibroma within the larynx is very uncommon. They represent 0.1% to 1.5% of all benign laryngeal tumors, schwannoma being slightly more frequent than neurofibroma [4]. 80% are located in the aryepiglottic fold, 20% in the false or true vocal cords [5-7]. They usually grow submucosal; with a few reports describing polypoid growth [6]. There seems to be a slight female preponderance [2,6]. The internal branch of the superior laryngeal nerve is most likely the nerve of origin [8,9].

## Case report

A 44-year-old woman was referred by her ENT physician for clinical diagnosis and treatment of a mass discernable endoscopically under the intact mucosa of her left false vocal cord. She had a 2- to 3-year history of hoarseness and dyspnoea on exertion with no complaint of dysphagia. She had a 15 pack-year smoking history. Further medical history was unremarkable, physical examination was normal.

**Figure 1:**
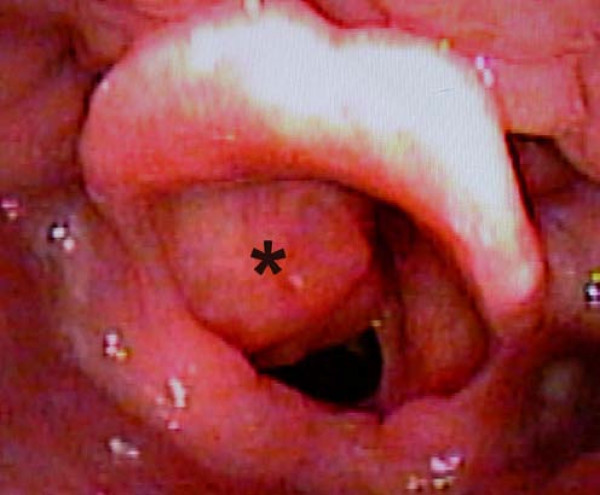
**Laryngoscopic view of the submucosal tumor in the left false vocal cord (asterisk)**. The view of the true vocal cord is blocked.

Fiberoptic laryngoscopy revealed a large submucosal mass within the left false vocal cord obstructing the view of the hypo-mobile true vocal cord (Fig. 1). The mobility of the right vocal cord was normal.

**Figure 2:**
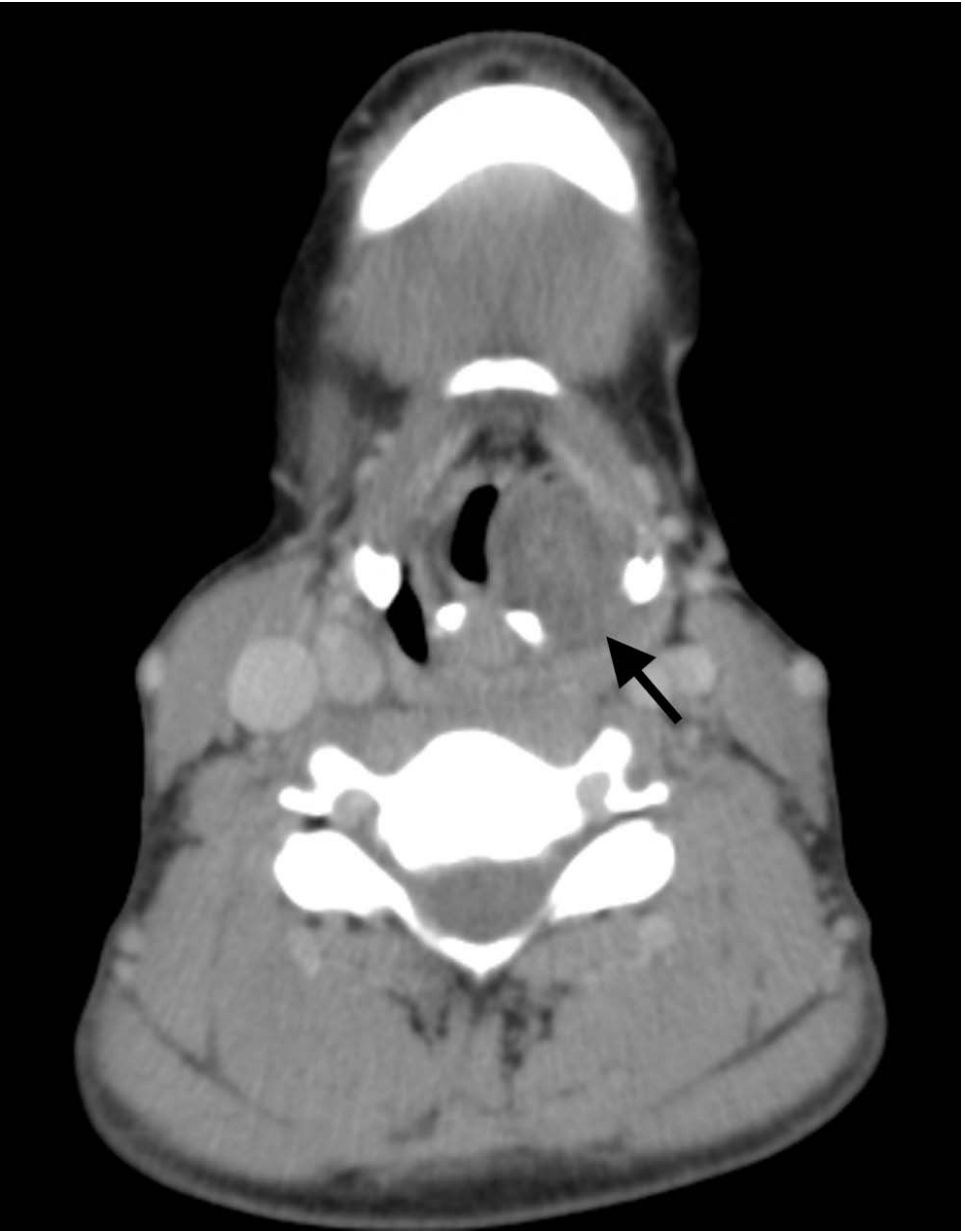
**CT image of the well defined, hypodense, round to oval submucosal mass in the left supraglottic larynx (arrow)**.

Computed tomography (CT) demonstrated a 22 × 23 × 28 mm well defined, round to oval, mass in the left supraglottic larynx growing under intact mucosa. Compared to muscle it was hypodense, slightly inhomogeneous with a clear capsule and no sign of infiltrative growth or cartilaginous destruction (Fig. 2).

**Figure 3:**
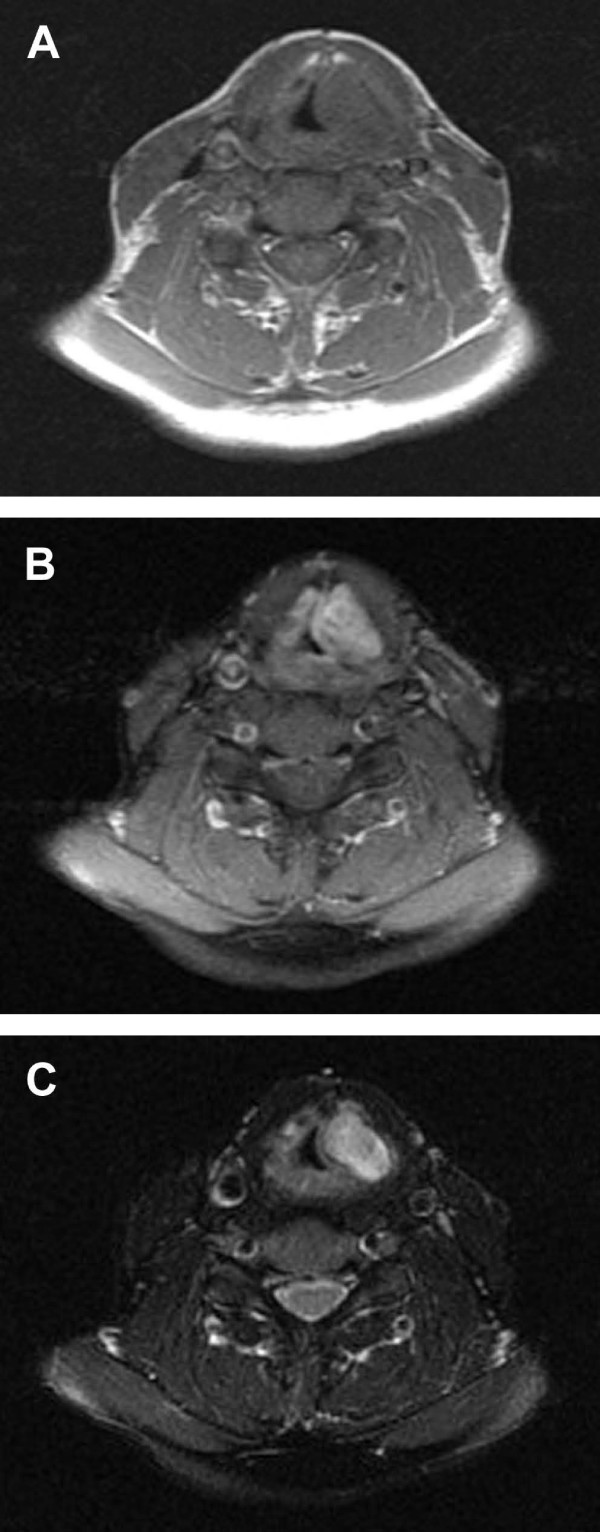
**MRI images of the supraglottic tumor**. (A) T1-weighted image without Gadolinium, (B) T1-weighted image with Gadolinium, (C) T2-weighted image.

Magnetic Resonance Imaging (MRI) of the lesion revealed it to be isodense compared to muscle in T1-weighted images with strong, inhomogeneous enhancement of Gadolinium. In T2, the lesion was hyperintense and also inhomogeneous. The lesion was well defined with no sign of infiltrative growth (Fig. 3).

Suspension microlaryngoscopy was performed under general anaesthesia, with trans-oral laser assisted incision biopsy. The supraglottic findings were consistent with the fiberoptic examination. The glottis and subglottis were normal. In frozen sections, the diagnosis was: benign mesenchymal tumor, probably schwannoma. To obtain a definite diagnosis, the tumor was resected as far as possible through the incision in the false vocal cord and sent for histopathological evaluation. The patient was extubated primarily and recovered well.

Meanwhile, MRI scanning of the neurocranium, cerebellopontine angle and spine yielded normal findings without signs of further nerve sheath tumors or Neurofibromas.

**Figure 4:**
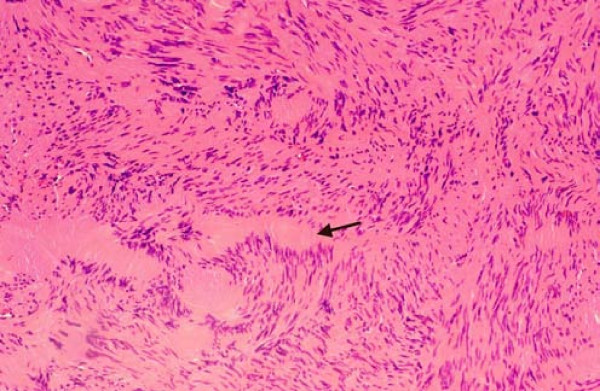
**Histologic appearance of the Antoni A regions with compact cell bundles and Verocay bodies (arrow)**. Hematoxylin-eosin, magnification ×100.

Definite histopathological evaluation confirmed the diagnosis of laryngeal schwannoma with low proliferative activity (Fig. 4).

Three months after initial surgery, the patient was re-admitted for persisting hoarseness, dyspnoea and sore throat. She still had no complaint of dysphagia. Fiberoptic laryngoscopy again showed fullness of the left false vocal cord with partial obstruction of the supraglottic airway. The left true vocal cord was immobile. CT scan demonstrated a 20 × 20 × 25 mm relapse of the well-defined, slightly inhomogeneous tumor in the left supraglottic larynx extending to the infraglottic space. The patient was taken back to the operative theatre for CO_2 _laser excision of the tumor. After surgery, the patient was extubated primarily and recovered well. She was discharged on postoperative day three. Histopathological evaluation confirmed the diagnosis of a relapse of the schwannoma that was resected three months earlier. The proliferative activity was still low with no sign of malignancy.

**Figure 5:**
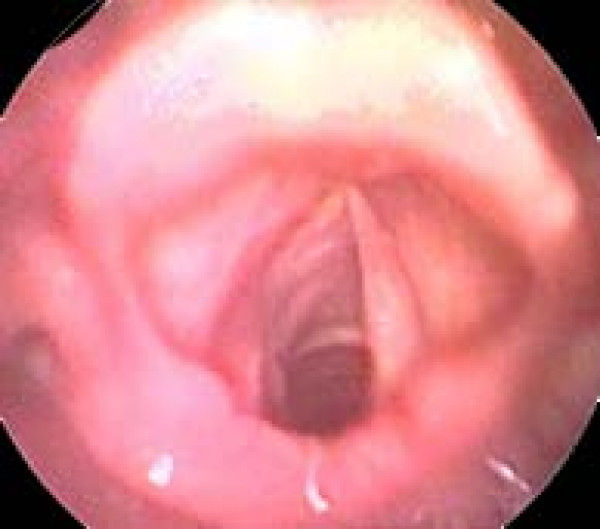
**Laryngoscopic view three months after endoscopic laser-resection of the tumor**.

The final examination three months after the second surgery revealed no sign of recurrence of the tumor (Fig. 5), the patient had recovered well free of complaints and was satisfied with her voice.

## Discussion

Two different types of neurogenic tumors of the larynx have been described: Schwannomas and neurofibromas. Both entities are rare and comprise only about 0.1% to 1.5% of all benign laryngeal tumors [4]. Neurofibromas are encountered more frequently in Neurofibromatosis. Malignant transformation is reported in 10% of neurofibromas while in schwannoma it is very uncommon [2]. Neurogenic tumors of the larynx are most frequently located in the aryepiglottic fold or in the true or false vocal cords [5-7].

The clinical symptoms of the disease are those usually associated with a slow growing lesion of the larynx: Over a period of years the patient gradually develops hoarseness, globus sensation, dysphagia, dyspnoea on exertion with inspiratory, sometimes biphasic stridor [2,6]. Some patients complain about dyspnoea in the supine position which seems to be associated with the location of the lesion [6,10]. One case of asphyxial death due to laryngeal schwannoma is reported [10].

The true vocal cord on the affected side is usually immobile or hypomobile [6,11,12], although some authors report normal mobility [9,13]. In many cases, such as in our reported case, the bulgy supraglottic tumor obstructs the view of the true vocal cord [14,15]. Also, the mass effect of a large tumor can mimic fixation of the cricoaritenoid joint ("pseudo-fixation") [6].

The diagnostic workup should include indirect and fiberoptic laryngoscopy which usually reveals a submucosal mass in the described location. Such a lesion coupled with impaired vocal cord mobility should draw attention towards a neurogenic tumor.

In CT scans of the disease, most authors describe a well defined, hypodense submucosal mass without signs of infiltrative or destructive growth [11,16-19]. Many authors describe a heterogeneous contrast enhancement [6,9,12,14,17,19,20].

On MRI scanning, the lesion is expected isointense to slightly hyperintense in T1-weighted images with strong, inhomogeneous enhancement of Gadolinium, in T2 the lesion is hyperintense [11,12,17,18,21].

The differential diagnoses of neurogenic tumors of the larynx include chondroma and adenoma [21]. Also laryngeal cyst and internal laryngocele should be taken into consideration [6].

A definite diagnosis can only be made histologically. Schwannomas almost exclusively are comprised of spindle cells with long, oval nuclei and indistinct cell membranes. These Schwann cells either form cellular regions with compact cell bundles with nuclei lining up in palisades (Antoni A regions) or edematous regions with loosely arranged cells in a myxoid matrix prone to degeneration (Antoni B regions) [2,6]. Two compact rows of well aligned nuclei separated by fibrillary cell processes are called Verocay bodies. Axons are usually not found in Schwannomas [6]. A clear capsule, the presence of Antoni A and/or Antoni B areas, and intense immunoreactivity for S-100 protein are criteria for the histologic diagnosis of Schwannoma [2,6].

Neurofibromas like Schwannomas exhibit an abnormal proliferation of Schwann cells. However, while Schwannomas emanate from Schwann cells, neurofibromas emanate from perineural fibrocytes. They are not encapsulated and comprise a variety of cell types: elongated spindled Schwann cells interwoven with axons and collagen fibers [4,6,22]. Thus an important feature of neurofibroma is entwining of the tumor with the parental nerve fascicles while Schwannoma grows extrinsic to the nerve fibers [6].

The surgical separation of the tumor from the nerve is theoretically possible in Schwannoma, whilst in neurofibroma it is impossible [4].

The only effective therapeutic option in benign neurogenic laryngeal tumors is complete resection. Since the diagnosis can only be made histologically, direct laryngoscopy with biopsy of the lesion will usually be the first step in treatment [6,13,18,23]. However, in Schwannoma biopsy can be difficult due to the solid capsule of the tumor [6,23]. Complete surgical excision of the tumor should be planned according to the individual requirements of each case. Most authors favor external approaches with alternative airway provisions such as a preliminary tracheotomy in larger tumors. Median or lateral thyrotomy or median or lateral pharyngotomy are recommended [2,9,11,12,14,16,24]. In smaller tumors, endoscopic (laser-assisted) resection of the tumor can be a reasonable treatment option [2,13,18]. Independent of the approach, restoration of the vocal cord mobility is possible even if it was immobile prior to surgery [12,13,17].

In some cases including ours, rapid regrowth after incomplete resection of laryngeal schwannomas has been described [6].

At least one case has been reported where histologically verified laryngeal schwannoma revealed an unknown Neurofibromatosis type II [17]. Thus, if histology points towards neurofibroma but also in Schwannoma, neurofibromatosis should be considered and physical examination should rule out café au lait spots and further neurinomas. Also, MRI should rule out neurinomas of the cranial nerves and spinal cord [17-19].

An association of benign solitary schwannomas with malignant tumors (especially skin and breast cancer) has been reported [25]. Dermatological and when appropriate gynecologic consultations should be considered.

## Summary

• Laryngeal schwannoma is a rare, slow growing, benign neurogenic tumor usually located in the supraglottic larynx, rarely in the glottis.

• It grows submucosal and is well encapsulated.

• It can cause globus sensation, dysphagia, dysphonia and upper airway obstruction.

• A definite diagnosis can only be made histologically.

• The only curative treatment option is complete surgical excision.

• Incomplete excision can result in rapid regrowth with life threatening complications.

• In cases of solitary schwannomas, Neurofibromatosis as well as malignant tumors especially of the skin and breast should be ruled out.

## Consent

Written informed consent was obtained from the patient for publication of this case report and accompanying images. A copy of the written consent is available for review by the Editor-in-Chief of this journal.

## Competing interests

The authors declare that they have no competing interests.

## Authors' contributions

JE performed the literature research, composed the manuscript and performed the surgeries. UR critically revised the manuscript. HBG performed the radiological studies, critically revised the manuscript with special regard to radiologic findings. UH performed and evaluated the histology, critically revised the manuscript with special regard to histologic findings. RM critically revised the manuscript. ME performed pre- and postoperative examinations of the patient, provided photographs, drafted a first version of the manuscript. TU critically revised the manuscript with special regard to language. HS supervised the work, performed the surgeries and critically revised the manuscript. All authors approved the final manuscript.
